# Editorial: Plant Epigenetics and Climate Change

**DOI:** 10.3389/fpls.2022.955159

**Published:** 2022-06-14

**Authors:** Naganand Rayapuram, Axel De Zelicourt, Santosh B. Satbhai, Mario Alberto Arteaga-Vazquez

**Affiliations:** ^1^Center for Desert Agriculture, King Abdullah University of Science and Technology, Thuwal, Saudi Arabia; ^2^Université Paris-Saclay, CNRS, INRAE, Université Evry, Institute of Plant Sciences Paris-Saclay (IPS2), Gif-sur-Yvette, France; ^3^Department of Biological Sciences, Indian Institute of Science Education and Research (IISER), Sahibzada Ajit Singh Nagar, India; ^4^Instituto de Biotecnología y Ecología Aplicada (INBIOTECA) de la Universidad Veracruzana, Xalapa, Mexico

**Keywords:** epigenetics, climate change, global warming, mangrove, heat stress

Our planet is warming up at an unprecedented rate in recorded history. Higher temperatures alter weather patterns and skew the natural equilibrium. All forms of life are at risk due to the emission of greenhouse gases mainly due to human activity. The primary culprits are increasing atmospheric carbon dioxide and the rise in global temperature that together result in climate-related constraints such as drought and heat waves. These factors have a significant negative impact on land and marine ecosystems. The genetic makeup of organisms is essential for sensing and responding to the ever-changing environmental conditions and there is mounting evidence pointing to a major role for epigenetic regulation as part of the adaptation strategies that allow organisms to respond to the selective pressure enforced by climate change.

Epigenetics study heritable changes in gene expression that do not involve modifications in the DNA sequence *per se* and arise in response to internal and external environmental cues. Organisms use epigenetic modifications as a means to transfer environmental information to their offspring. While the plasticity of the genome has been widely described, the mechanisms that propel populations toward variations in phenotypes are still being elucidated. On the other hand, climate change constitutes one of the biggest challenges that plant scientists have been facing with the need of increasing crop yields in an uncertain environment. The objective of this Research Topic is to bring together research in two fields: climate change and epigenetics, aiming to uncover epigenetic processes underlying acclimation and adaptation of plants to climate change toward finding agronomic solutions to meet the ever-rising worldwide demand for food.

This special issue on “*Plant Epigenetics and Climate Change*” is a small bouquet of research and review articles that provides new perspectives and broad overviews on the impact of environmental cues related to climate change in defining epigenetic modifications and how these modifications, in turn, can be exploited to tailor crop plants to overcome the threat to food production.

Mangroves are prolific ecosystems. They serve a range of biological and socioeconomic roles. Indeed, mangrove trees fix up to 100 times more carbon than terrestrial forests and they do it in a more permanent manner. Therefore, they offer a unique and highly efficient way to mitigate climate change. Individual trees in mangroves have such little genetic variety that they are virtually indistinguishable from one another, and they are believed to rely on epigenetic variation to adapt to environmental changes. In a brief review, Miryeganeh summarizes current findings on epigenetic regulation and adaptability in mangroves. On similar lines, in another thought-provoking review on the climate change-induced decline of forests, García-García et al. discuss the vulnerability of forest tree species to changing climatic conditions. They further shed some light on the challenges faced by researchers such as huge genome sizes, the lack of reference genomes and annotations, to study the epigenetic mechanisms used by them to adapt to the ever-changing environmental conditions that result in the decline of forest cover.

Cotton, an economically important crop, is well-known for its tolerance to saline and sodic soils. However, adverse climatic conditions severely affect the growth and yield of cotton. With a broad objective to aid cotton breeders to develop cotton varieties that are well-adapted to harsh environmental conditions, Rui et al. generated a methylome map using Whole Genome Bisulfite Sequencing (WGBS) of *Gossypium barbadense*. They used this data to understand the epigenetic mechanisms employed to contend with salt and alkaline stresses.

Crop yields are predicted to decrease by one-third by 2050 because of global warming. We require plants that can withstand higher temperatures, blossom, and grow for a longer period of time. The ultimate objective is to be able to change temperature responsiveness to secure the future of our food supply. Repeated exposure of plants to heat stress results in significantly higher tolerance to heat. Yadav et al. exposed Arabidopsis plants to heat stress for 25 consecutive generations and showed that repeated exposure of the plants to heat stress resulted in higher number of mutations in the form of single nucleotide polymorphisms (SNPs), insertions and deletions (INDELs). When they looked at the methylome of heat stressed plants they exhibited a higher frequency of methylation changes in gene bodies relative to a lower methylation in the body of transposable elements (TEs). These findings can be utilized to develop thermo-tolerant crop breeding programs.

Under adverse conditions, plants compromise on their growth and channelize their efforts to activate stress response to overcome the stressful conditions. There is growing evidence that target of rapamycin (TOR) signaling pathway plays an important role in stress signaling. The work by Sharma et al., sheds some light on the molecular architecture by which glucose-TOR signaling combines stress and energy signaling to regulate thermotolerance *via* distinct modules.

This collection of research articles and reviews shows the application of avant garde technologies to study the convergence of diverse fields such as climate change and plant epigenetics as a way to lay a solid framework aiming to tackle the problem of food security under global warming conditions.

## Author Contributions

NR, AD, SS, and MA-V proposed the Research Topic, edited manuscripts, and wrote the editorial. All authors contributed to the article and approved the submitted version.

## Conflict of Interest

The authors declare that the research was conducted in the absence of any commercial or financial relationships that could be construed as a potential conflict of interest.

## Publisher's Note

All claims expressed in this article are solely those of the authors and do not necessarily represent those of their affiliated organizations, or those of the publisher, the editors and the reviewers. Any product that may be evaluated in this article, or claim that may be made by its manufacturer, is not guaranteed or endorsed by the publisher.

